# Analysis of decomposition in 23 seafood products by liquid chromatography with high‐resolution mass spectrometry with sensory‐driven modeling

**DOI:** 10.1002/fsn3.2223

**Published:** 2021-03-11

**Authors:** Randy L. Self, Michael G. McLendon, Christopher M. Lock, Jinxin Hu

**Affiliations:** ^1^ Pacific Northwest Laboratory Office of Regulatory Affairs U.S. Food and Drug Administration Bothell WA USA

**Keywords:** LC‐MS, multivariate analysis, Seafood, Sensory

## Abstract

Samples of 23 seafood products were obtained internationally in processing plants and subjected to controlled decomposition to produce seven discrete quality increments. A sensory expert evaluated each sample for decomposition, using a scale of 1–100. Samples were then extracted and analyzed by liquid chromatography with high‐resolution mass spectrometry (LC‐HRMS). Untargeted data processing was performed, and a sensory‐driven Random Forest model in the R programming language for each product was created. Five samples of each quality increment were analyzed in duplicate on separate days. Scores analogous to those obtained through sensory analysis were calculated by this approach, and these were compared to the original sensory findings. Correlation values (r) were calculated from these plots and ranged from 0.971 to 0.999. The finding of decomposition state of each sample was consistent with sensory for 548 of 550 test samples (99.6%). Of the two misidentified samples, one was a false negative, and one false positive (0.2% each). One additional sample from each of the 1st, 4th, and 7th increments of each product was extracted and analyzed on a third separate day to evaluate reproducibility. The range of these triplicate calculated scores was 15 or less for all samples tested, 10 or less for 63 of the 69 triplicate tests (91%), and five or less for 41 (59%). From the models, the most predictive compounds of interest were selected, and many of these were identified using MS^2^ data with standard or database comparison, allowing identification of compounds indicative of decomposition in these products which have not previously been explored for this purpose.

## Graphical abstract

This work involves a new analytical method for the determination of seafood decomposition in 23 products. LC‐HRMS was used in conjunction with sensory analysis to create models capable of producing sensory‐like outcomes using instrumentation. By doing so, we hope to provide a better compliment to sensory than existing techniques.

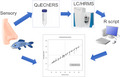

## 1 INTRODUCTION

The United States Food and Drug Administration (USFDA) routinely samples and tests seafood products for decomposition, or spoilage, to ensure consumer safety and sanitary production conditions (USFDA, 2010). Samples are analyzed primarily by sensory analysis according to guidelines developed in conjunction with the Canadian Food Inspection Agency (CFIA) and the National Oceanic and Atmospheric Administration (NOAA) (FAO, 1999). However, a major drawback to sensory is the extensive training required (ASTM, 1981; Labbe et al., 2004; USFDA, 2013). Uniformity of testing results is also a potential concern (Næs, 1990; Rossi, 2001; Wilkinson & Yuksel, 1997). Alternative testing procedures currently in use are limited to analysis of histamine (AOAC, 1987) or indole (AOAC, 1982). Histamine content is typically only useful for particular products (Hungerford, 2010), and indole is indicative only of warmer temperature decomposition in shrimp (Chang et al., 1983). Since neither of these measures decomposition in a way that can be directly compared with sensory results, there is a need for an alternative technique.

Other existing techniques to measure particular chemical indices of decomposition in various seafood products include trimethylamine (Leroi et al., 2001), other biogenic amines (Emborg et al., 2002; Self et al., 2011), and broader selections of volatiles (Bai et al., 2019; Jaffrès et al., 2011; Joffraud et al., 2001; Jørgensen et al., 2001). Less‐targeted techniques have also been explored, including “electronic nose” style devices (Du et al., 2001; Lim et al., 2013; Lv et al., 2018), analysis of bioelectrical impedance (Fan et al., 2021; Sun et al., 2020), and, more recently, mass spectrometry‐based computer modeling (Kuuliala et al., 2018; Mikš‐Krajnik et al., 2016), which is the focus of the current work.

Following an initial proof of concept limited to six species of salmon (Self et al., 2019), there has been Congressional interest in sensory alternatives for regulatory use (U. S. Congress, 2018). This has enabled the current study, which improves and greatly expands on that previous work.

## 2 MATERIALS AND METHODS

### 2.1 Experimental overview

This study involves the use of sensory‐driven computer models based on liquid chromatography with high‐resolution mass spectrometry (LC‐HRMS) data for 23 internationally sourced seafood products. Samples were first analyzed by sensory and then immediately stored in −20°C (±2°C) conditions until further use. These were subsequently thawed, homogenized, and extracted using a modified “Quick, Easy, Cheap, Effective, Rugged, and Safe” (QuEChERS) technique (Anastassiades et al., 2003). Extracts were analyzed with LC‐HRMS, with untargeted data processing.

Sample responses were used in conjunction with sensory data to create statistical models. This enables the calculation of a “decomposition score” which is analogous to that generated by sensory analysis. These model‐produced scores were then compared with the original sensory data.

### 2.2 Equipment and reagents

#### 2.2.1 Extraction equipment

Samples were comminuted / homogenized using either a Robot Coupe RSI 2Y‐1 (Robot Coupe, Ridgeland, MS, USA) mixer or consumer‐grade KFC3100 food processor (KitchenAid, Benton Harbor, MI, USA). Extractions were carried out using 50‐mL and 1.5‐mL conical polypropylene centrifuge tubes (Thermo Fisher Scientific, Waltham, MA, USA). Shaking steps employed a Wrist Action™ model 75 shaker (Burrell Scientific, Pittsburgh, PA, USA) and a Vortex Genie 2® vortex mixer (Scientific Industries Bohemia, NY USA). High‐purity water used in the extraction and instrumental mobile phase was produced by a Milli‐Q® purification system with an LC‐Pak® polisher (MilliporeSigma, Burlington, MA, USA). The initial centrifugation step used an IEC PR7000 M centrifuge (Thermo Scientific, Waltham, MA, USA), and subsequent cleanup steps used a model 5,418 centrifuge (Eppendorf, Hamburg, Germany). Titan 2™ (17 mm diameter, 0.45‐µm pore size) nylon syringe filters (SUN Sri, Rockwood, TN, USA) with 1‐mL polypropylene syringes (Becton, Dickinson & Co. Franklin Lakes, NJ, USA) were used to filter the final extract into 2‐mL amber glass injection vials with 200‐µL glass inserts (Thermo Fisher).

#### 2.2.2 Reagents

Dry ice (Airgas, Woodinville, WA, USA) was used in some homogenization procedures. LC/MS grade acetonitrile (Fisher Scientific, Hampton, NH, USA) and pouches containing 1.5 g of NaCl and 6 g of anhydrous MgSO_4_ (United Chemical Technologies, Bristol, PA, USA) were used in the initial QuEChERS extraction. Polypropylene tubes (2 ml) containing 50 mg primary–secondary amine sorbent and 150 mg MgSO_4_ (Agilent Technologies, Santa Clara, CA, USA) were used in the cleanup step. LC‐MS grade methanol and formic acid (Fisher) were used in LC mobile phase. Ethyl docosahexaenoate, N‐acetylhistamine, 5‐methylcytosine hydrochloride, tyramine, thymine, choline chloride, N‐acetyltyramine, N‐acetylcadaverine, 3‐methyladenine, N‐acetylhistamine, and creatinine (Fisher); N‐acetylputrescine hydrochloride (Sigma‐Aldrich, St. Louis, MO, USA); and 3‐methylguanine (Toronto Research Chem., North York, ON, Canada) were used as standards for identity confirmation.

#### 2.2.3 Instrumental equipment

Samples were analyzed using an UltiMate 3,000 HPLC (Dionex, Sunnyvale, CA, USA), with an Acquity UPLC® BEH C18 column (2.1 x 100 mm, 1.7‐µm pore size) (Waters, Milford, MA, USA). This was interfaced to a Q‐Exactive HF™ mass spectrometer (Thermo Scientific, Bremen, Germany).

### 2.3 Sampling and sensory analysis

#### 2.3.1 Sample collection and development

Sampling teams, each led by a USFDA certified National Seafood Sensory Expert (NSSE), were deployed to seafood packing facilities in Kodiak, Alaska (USA); Guayaquil, Ecuador; Georgetown, Guyana; and Huy Toa, Vietnam, to collect samples of 23 seafood products (Table 1). Products were sampled in the freshest possible state and subjected to controlled decomposition on ice onsite. Samples were removed from ice at sensory‐controlled timepoints to create discrete quality increments. The general strategy was to create seven such increments, ranging from the freshest available (1) to very advanced decomposition (7). However, eight such increments were collected for swordfish (without CO), and nine for canned tuna, at NSSE discretion. Canned tuna samples were then canned, and all others were vacuum‐sealed and stored at −20°C (±2°C) until further use. Five sample portions (approximately 200 g) of each increment were used in the study.

**TABLE 1:** List of products sampled

Abbreviation	Common name	Scientific name	Form	Origin
CHUM	Chum salmon	*Oncorhynchus keta*	Skin‐on filet	Alaska
COHO	Coho salmon	*Oncorhynchus kisutch*	Skin‐on filet	Alaska
CRK	Croaker	*Micropogonias furnieri*	Skin‐on filet	Guyana
ESCO	Escolar	*Lepidocybium flavobrunneum*	Skin‐off filet	Ecuador
GRCO/GRNCO^a^, ^b^	Grouper	*Epinephelus areolatus*	Skin‐off filet	Vietnam
MCO/MNCO^a^	Mahi mahi	*Coryphaena hippurus*	Skin‐off filet	Ecuador
POP	Pacific Ocean Perch	*Sebastes alutus*	Skin‐off filet	Alaska
SCAL	Peruvian scallop	*Argopecten purpuratus*	Shucked, raw, roe‐off	Ecuador
PINK	Pink salmon	*Oncorhynchus gorbuscha*	Skin‐on filet	Alaska
POL	Pollock	*Gadus chalcogrammus*	Skin‐on filet	Alaska
RSNP	Red snapper	*Lutjanus campechanus*	Skin‐on filet	Guyana
SHRP	Shrimp	*Litopenaeus vannamei*	Raw, headless, shell on	Ecuador
SNP^b^	Snapper	*Lutjanus sanguineus*	Skin‐off filet	Vietnam
SOCK	Sockeye salmon	*Oncorhynchus nerka*	Skin‐on filet	Alaska
SQD^c^	Squid	*Loligo spp*.	Tubes and tentacles	Vietnam
SFCO/SFNCO^a^	Swordfish	*Xiphias gladius*	Skin‐off steaks	Vietnam
WEAK	Weakfish	*Cynoscion regalis*	Skin‐off filet	Guyana
YFCO/YFNCO^a^	Yellowfin tuna	*Thunnus albacares*	Skin‐off steaks	Vietnam
YFCAN	Yellowfin tuna	*Thunnus albacares*	Canned in broth	Ecuador

^a^ Carbon monoxide treated (CO) and nontreated (NCO).

^b^ Aquacultured products.

^c^ Mixed spp. in genus.

#### 2.3.2 Sensory analysis

Following official policy implemented in USFDA regulatory laboratories (USFDA, 2010), sensory evaluations were made using a single, highly qualified expert (NSSE) in lieu of a sensory panel. Assessments were made according to established procedures (FAO, 1999), based on both quality and intensity of odor characteristics. In this way, a numerical score was assigned on a 100‐point scale, with greater values indicative of lower quality. Scores below 50 are considered nondecomposed, and greater than 50 decomposed. For samples consisting of many small pieces (e.g., scallops, shrimp, squid), assessments were made for each sample in bulk, and care was taken to remove any strongly outlying pieces, although this was rare.

### 2.4 Extraction

Samples of scallops, shrimp (peeled), and squid were added to an approximately equal mass of dry ice and blended to a powdery consistency in an industrial‐grade blender as described in the literature (Bunch et al., 1995) and stored overnight at −20°C to allow CO_2_ to sublimate. All other samples were skinned if present, then blended to a uniform consistency using a consumer‐grade food processor. Samples of these composites (10.0 ± 0.5 g) were transferred to 50‐mL centrifuge tubes and extracted via a QuEChERS (Anastassiades et al., 2003) technique, modified as in previous work (Self et al., 2019). Resulting extracts were mixed with an equal volume of high‐purity deionized water and filtered via a 0.45‐µm syringe filter prior to analysis. Extraction was performed in duplicate on separate days for all samples, using the same composite. Additionally, a third replicate on a third day was prepared for one sample each from the 1st, 4th, and 7th (or highest) sets (low, borderline, and high decomposition states) of each product to establish reproducibility across the range.

### 2.5 LC‐HRMS analysis

#### 2.5.1 LC conditions

Separation conditions are as described in previous work (Self et al., 2019) and here briefly summarized. Aqueous (A) or methanolic (B) solutions of 4mM ammonium formate with 0.1% formic acid were the mobile phase components. A starting condition of 95%/5% (A/B) was held two minutes, followed by a linear ramp to 5%/95% (A/B) at 28 min, held until 33 min. The column temperature was held at 40°C, and sample vials were held at 10°C. The injection volume was decreased to 1 µL.

#### 2.5.2 HRMS conditions

Positive mode electrospray ionization (ESI) was used, with a probe temperature of 400°C, at position C,1.0,0. The spray voltage was 3.00 kV, inlet capillary temperature was 380°C, and the S‐lens RF level was 65.0. Sheath, auxiliary, and sweep gasses were set to 60, 30, and 10 units of N_2_, respectively. A full MS scan was taken with the 60,000‐resolution setting, followed by an all‐ions fragmentation (AIF) scan, at 30,000 resolution, using normalized collision energies (NCE) of 10, 40, and 60. Each of these scanned from m/z 80–1200 with an automatic gain control (AGC) target of 3 × 10^6^ and maximum injection time of 200 ms. Additional analysis was performed on selected samples and standards to assist with compound identification, which employed the same full scan settings with data‐dependent MS^2^ (DDMS2) and NCE as described for the AIF scan. These either included a target list of interest or targeted the top five ions in each scan, as needed.

### 2.6 Data processing and analysis

#### 2.6.1 Untargeted analysis

Sample data were subjected to an untargeted workflow using Compound Discoverer® 3.1, which included retention time alignment, grouping, feature merging, and gap filling. Each product was treated separately, and samples were segregated between decomposed, with sensory scores greater than 50, and nondecomposed, with scores less than 50. Differential analysis was performed using a volcano plot, with a log_2_ fold change greater than one, and compounds with the 100 lowest *p*‐values were selected to find those most relevant to decomposition.

#### 2.6.2 Modeling

Peak area data for the 100 compounds of interest for samples in each product set were exported from Compound Discoverer and combined with the original sensory score data. These were incorporated into a script in the R (3.6.3) programming environment. One third of the samples, rounding up, were randomly selected to be the test set, using a “set.seed” value to ensure reproducibility, while the remaining samples comprised the training set. A data matrix for each product was formed using peak area counts for each compound as dependent variable columns, sensory scores as the independent variable column, and one row for each training sample. These matrices were then subjected to the Random Forest algorithm (Breiman, 2001), using regression with 2000 trees and fitting to the sensory data. This initial model was used to evaluate the predictive power of each compound and optimize the number of compounds used for further modeling. Compounds were ranked by predictive power, and then, the number of compounds used to model was iterated and optimized with respect to correlation values (r) between sensory and modeled scores. This optimized compound list for each product (Table 3) was then used to generate a second Random Forest model which was used to predict sensory‐like scores for each sample in the test set of each product.

#### 2.6.3 Compound identification

Samples containing high levels of compounds in the optimized compound lists were subjected to DDMS^2^ analysis as described in section 2.5.2 above. This dataset was subjected to an additional Compound Discoverer analysis, incorporating searches using the Chemspider© (Royal Society of Chemistry, 2020) and mzCloud™ (HighChem LLC, 2020) databases. Sample data were compared with these sources and in silico fragmentation using the fragment ion search (“FISh scoring”) technique to find putative best match candidates for each compound of interest. Reference standards were then purchased as available. These were diluted to approximately 1 µg/mL in 1:1 (v:v) methanol:water and analyzed using the same instrumental techniques for confirmation. Subsequent spectral confirmation for other compounds utilized mzCloud or METLIN (Guijas et al., 2018) databases.

## 3 RESULTS AND DISCUSSION

### 3.1 Method performance

#### 3.1.1 Accuracy

Regulatory sensory analysis is reported as a qualitative pass/fail finding (USFDA, 2010), but the scoring technique as described in section 2.3.2 above is useful in training and for comparison between analysts. These sensory scores were used to train the models and are also useful in evaluating the modeling method, although this should also be considered a primarily qualitative technique.

**FIGURE 1:**
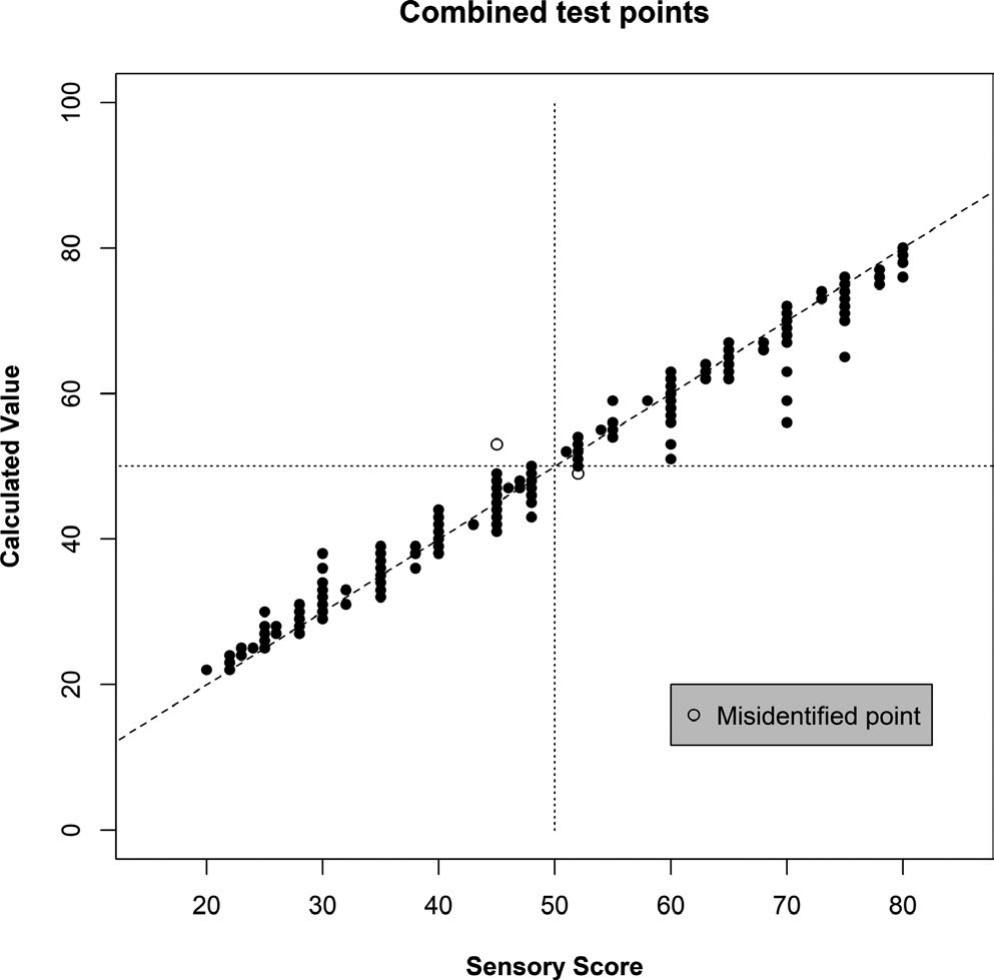
Combined correlation plot of test data points for all products

Correlation (r) between model‐produced and sensory values was calculated for each product test set. These ranged from 0.971 to 0.999 (Table 2). A false‐positive finding was defined as any finding for which the sensory score was less than 50 (i.e., nondecomposed) while the calculated value was greater than 50 (decomposed). A sensory score greater than 50 with a calculated score less than 50 was likewise considered a false‐negative finding. One of each of these was found in all test samples (Figure 1), corresponding to an overall false‐positive and false‐negative rates of 0.2% (*n* = 550) each. The false‐positive finding in the Coho salmon set arose from a modeled value of 53 compared with the original sensory score of 45. For Pacific Ocean perch, a modeled value of 49 compared with the sensory value of 52 generated a false‐negative finding. For each of these cases, the values are quite close to the dividing line of 50 and close to the sensory findings. This indicates that the method is quite reliable, with some care taken with results in this range.

**TABLE 2:** Accuracy summary

Product	Samples	Test Samples	Correlation
CHUM	73	24	0.995
COHO	72	24	0.981
CRK	73	24	0.971
ESCO	73	24	0.995
GRCO	73	24	0.999
GRNCO	71	24	0.988
MCO	73	24	0.996
MNCO	73	24	0.999
PINK	56	19	0.998
POL	73	24	0.995
POP	70	23	0.995
RSNP	73	24	0.997
SCAL	73	24	0.998
SFCO	73	24	0.983
SFNCO	83	28	0.997
SHRP	73	24	0.997
SNP	73	24	0.994
SOCK	73	24	0.990
SQD	73	24	0.999
WF	73	24	0.971
YFCAN	72	24	0.998
YFCO	73	24	0.996
YFNCO	73	24	0.997

#### 3.1.2 Reproducibility

**FIGURE 2:**
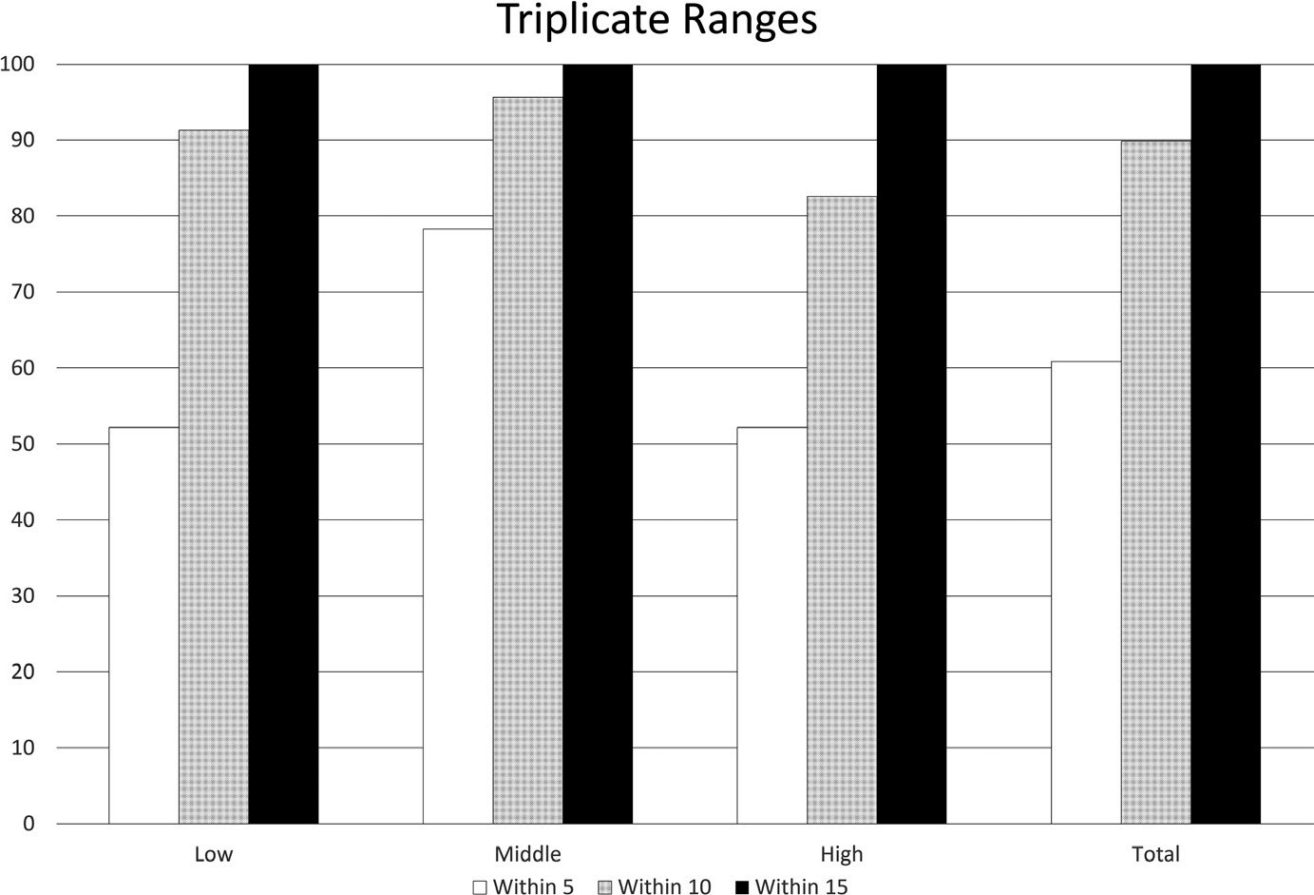
Ranges of triplicate data, showing ratio within 5, 10, and 15 sensory score points for low, middle, and high decomposition states for all products

As discussed in section 2.4 above, triplicate analysis was performed for one sample from low, borderline, and high decomposition sets for each product. Scores were calculated for each of these samples, whether they were randomly selected for the test set or not. Reproducibility of the technique was evaluated by examining the overall point score range of these triplicate analyses (Figure 2).

All ranges for these triplicate measurements (*n* = 69) were less than 15 sensory score points, with 90% within 10 points and 61% within five points. Ranges were similar for low, middle, and high decomposition states, but trended somewhat lower for the middle state. Overall, it appears that reproducible results were generated by this technique as demonstrated by these data.

### 3.2 Compound identification

As shown in Table 3, the putative identities of 13 compounds of interest were confirmed using reference standards as described in section 2.6.3 above. These were confirmed by comparing sample peak retention time (RT, ±0.2 min), parent ion mass accuracy (±5 ppm), and that of at least one structurally significant fragment ion (±5 ppm), to those of the standard, as described in SANCO guidelines for pesticide analysis (SANCO, 2013). The identifications of an additional 14 compounds were confirmed by comparing the parent ion mass to calculated values, and fragment ion masses to spectral databases, within the same specifications. These should still be considered putative findings, as closely related or isomeric compounds may be responsible, and in some cases, cis/trans or stereoisomer conformations may be unknown. No confirmatory techniques were available for an additional 85 compounds, and other techniques will be required to further elucidate these.

**TABLE 3:** List of compounds used in modeling

Compound	Mass	RT	Confirmation	Used in models
3‐Methyladenine	149.07015	0.74	Standard	GRCO, RSNP
3‐Methylguanine	165.06506	1.35	Standard	CHUM
5‐Methylcytosine	125.05891	0.66	Standard	CHUM, PINK, POL
Acetylagmatine	172.13240	0.84	Standard	SQD
Acetylhistamine	153.09020	0.79	Standard	YFCAN
Acetylputrescine	130.11060	0.70	Standard	SQD
Acetyltyramine	179.09460	0.81	Standard	YFCAN
Acetylcadaverine	144.12630	0.84	Standard	CHUM, CRK, GRCO, GRNCO, MCO, POL, RSNP, SCAL, SFCO, WEAK
Choline	104.10750	0.82	Standard	MCO
Creatinine	113.05890	0.61	Standard	RSNP, SQD
Ethyl docosahexaenoate	356.27150	29.75	Standard	CRK, MCO, POP, WEAK
Thymine	126.04290	1.66	Standard	CRK, GRCO, POP, PINK, RSNP, WEAK
Tyramine	137.08410	1.40	Standard	CHUM, COHO, GRCO, GRNCO, MCO, MNCO, POL, POP, RSNP, SCAL, SFCO, SFNCO, SHRP, SNP, YFCO, YFNCO
1‐(Beta‐D‐Ribofuranosyl)−1,4‐dihydronicotinamide	256.10624	2.02	Database	MNCO, POL
18‐Phenyloctadecanoic acid	360.30306	30.90	Database	SNP
1‐Acetylpiperidine	127.09982	0.88	Database	MNCO, POL
1‐Phenyl−1,3‐octadecanedione	358.28741	30.28	Database	CRK
2‐Arachidonoylglycerol^a^	378.27736	27.43	Database	ESCO
2‐Methylbutyroylcarnitine	245.16310	6.76	Database	MNCO
7‐Aminomethyl−7‐deazaguanine	179.08118	3.29	Database	GRCO, RSNP, SQD, YFCO, YFNCO
7Z, 10Z, 13Z, 16Z, 19Z‐docosapentaenoic acid	330.25507	29.32	Database	SFNCO, SHRP, YFNCO
Adenosine	267.09692	2.35	Database	WEAK
Adrenic acid	332.27066	29.84	Database	SFNCO, SHRP
L−2‐Succinylamino−6‐oxoheptanedioic acid	289.07894	2.57	Database	POL
Plastoquinol−1	344.27173	29.72	Database	COHO
Retinal	284.21392	29.35	Database	SHRP, WEAK
Tryptophanamide	203.10642	7.44	Database	YFCAN
UKN001	101.03986	3.85	Unknown	COHO
UKN002	102.04785	3.85	Unknown	COHO
UKN003	104.06288	3.86	Unknown	COHO, SCAL, SFNCO
UKN004	113.08452	0.70	Unknown	SQD
UKN005	121.08955	3.89	Unknown	SQD
UKN006^a^	122.04971	1.39	Unknown	GRNCO
UKN007	126.04360	3.66	Unknown	PINK
UKN008	126.07961	0.77	Unknown	SCAL, YFNCO
UKN009	143.07364	6.00	Database	MNCO, SCAL, WEAK
UKN010	150.01412	5.22	Unknown	COHO
UKN011^a^	150.13678	0.60	Unknown	SCAL, SHRP
UKN012	163.08624	3.91	Unknown	POL
UKN013	172.03546	0.62	Unknown	PINK
UKN014	178.11101	10.68	Unknown	GRCO
UKN015	182.10594	6.78	Unknown	MNCO
UKN016^a^	183.06621	25.91	Unknown	CRK
UKN017^a^	189.02484	7.29	Unknown	WEAK
UKN018	195.07210	5.22	Unknown	COHO
UKN019	199.00599	1.44	Unknown	RSNP
UKN020	200.12024	29.78	Unknown	SQD
UKN021	209.12797	0.83	Unknown	ESCO
UKN022^a^	210.10076	3.37	Unknown	ESCO
UKN023	214.17231	29.81	Unknown	SQD
UKN024	226.13605	29.82	Unknown	SQD
UKN025	230.14249	8.07	Unknown	POL
UKN026	240.15155	29.82	Unknown	SQD
UKN027	244.06953	0.85	Unknown	POL
UKN028	250.05751	1.62	Unknown	PINK
UKN029	250.10680	0.84	Unknown	YFCAN
UKN030	253.24109	25.23	Unknown	YFCO
UKN031	264.07308	3.66	Unknown	PINK, RSNP
UKN032	274.10303	2.86	Unknown	SCAL
UKN033	278.08816	1.99	Unknown	SFCO
UKN034	294.05967	1.09	Unknown	YFCO
UKN035	302.28228	28.26	Unknown	MNCO
UKN036	306.12382	22.05	Unknown	GRCO
UKN037	309.30354	28.25	Unknown	SNP
UKN038	310.22934	29.75	Unknown	CRK, MCO, SHRP
UKN039	312.31434	24.49	Unknown	CHUM
UKN040	313.09953	0.63	Unknown	POP
UKN041	317.21089	5.09	Unknown	YFCAN
UKN042	321.27869	24.24	Unknown	PINK
UKN043	323.31920	28.65	Unknown	SNP
UKN044	325.27205	17.97	Unknown	WEAK
UKN045^a^	326.28172	32.87	Unknown	SHRP
UKN046	334.29853	23.14	Unknown	CHUM
UKN047	335.99712	1.08	Unknown	PINK
UKN048	336.08988	1.36	Unknown	WEAK
UKN049	336.08994	1.16	Unknown	SCAL, WEAK, YFCAN
UKN050	339.13353	18.04	Unknown	WEAK
UKN051	340.34595	28.29	Unknown	GRCO
UKN052	348.18378	30.88	Unknown	YFCO
UKN053	348.18382	28.26	Unknown	SOCK
UKN054	349.30961	28.12	Unknown	YFNCO
UKN055	349.30974	26.05	Unknown	YFCAN
UKN056	352.23723	29.33	Unknown	SFNCO
UKN057^a^	352.26123	26.66	Unknown	SFCO
UKN058	354.25377	29.82	Unknown	SOCK
UKN059	357.30337	30.75	Unknown	GRNCO
UKN060	357.97197	1.69	Unknown	RSNP
UKN061	365.29323	27.49	Unknown	CRK
UKN062	371.31917	31.61	Unknown	GRNCO
UKN063	375.32459	26.47	Unknown	ESCO, MCO, PINK, SOCK, YFNCO
UKN064	378.25372	29.80	Unknown	SOCK
UKN065^a^	384.19021	17.27	Unknown	SFNCO
UKN066	394.22780	29.83	Unknown	SOCK
UKN067	395.29399	26.82	Unknown	YFCO, YFNCO
UKN068	397.30964	27.75	Unknown	YFCO, YFNCO
UKN069	398.24385	29.11	Database	GRNCO
UKN070	400.29738	28.78	Unknown	MCO
UKN071	401.26253	27.23	Unknown	SFCO
UKN072^a^	404.27146	28.15	Unknown	ESCO
UKN073	408.32143	29.25	Unknown	SFCO
UKN074	413.13304	0.64	Unknown	POP
UKN075	421.30948	27.78	Unknown	YFNCO
UKN076	421.31030	25.71	Unknown	PINK
UKN077	423.32527	28.61	Unknown	YFNCO
UKN078^a^	437.40976	1.80	Unknown	YFCAN
UKN079	445.30009	29.92	Unknown	GRNCO
UKN080	447.31323	27.66	Unknown	MCO
UKN081^a^	531.43230	32.12	Unknown	CRK
UKN082^a^	546.33623	29.35	Unknown	CRK
UKN083^a^	631.28641	25.92	Unknown	CHUM
UKN084^a^	777.53040	31.99	Unknown	CRK
UKN085^a^	853.56261	32.72	Unknown	CRK

^a^ Compound detected at higher levels in nondecomposed samples. All others were higher in decomposed samples.

#### 3.2.1 Biogenic amine‐related compounds

Biogenic amines, with their relatively high polarity and small masses, present challenges for reverse‐phase liquid chromatography. It is therefore not surprising that of the amines commonly associated with seafood decomposition (Self et al., 2011), only tyramine was observed as a compound used in models. The N‐acetylated analogs of tyramine and several other compounds were detectable, however, as these are generally less polar than their base counterparts. Since no source of acetylation was added during the extraction, these likely arise from natural metabolic or fermentation processes. With more reliable chromatographic options, these compounds may also represent an interesting novel approach to characterization of biogenic amines in seafood decomposition in future work.

#### 3.2.2 DNA‐related compounds

DNA nucleosides were contributors to models in both free and modified forms. The only free base used in modeling was thymine, whereas methylated forms of adenine, cytosine, and guanine were used. Each of these was confirmed with standard analysis. While the exact mechanism of generation for these compounds was not explored, DNA degradation would seem most likely. There was also a database‐driven match for 7‐aminomethyl‐7‐deazaguanine, which is a known product of purine metabolism (Kanehisa & Goto, 2000).

Another potentially related database match was adenosine, although there are other potential sources for this. While DNA degradation has previously been used in a similar way to assess poultry spoilage (Faullimel et al., 2005), this appears to be a novel application of post‐degradation analytes in this way.

#### 3.2.3 Other compounds of interest

Lipids of various forms were major contributors to models and are primarily more prevalent in decomposed products. These vary extensively by product, likely due to natural differences in their initial makeup. Choline, a product of lipid metabolism (Kanehisa & Goto, 2000), was also identified.

In addition to the biogenic amine‐related compounds described above, other products of amino acid metabolism were also used in models. These include creatinine, a product of the same arginine, and proline metabolic process responsible for putrescine production (Kanehisa & Goto, 2000). Other examples include 1‐(beta‐D‐ribofuranosyl)‐1,4‐dihydronicotinamide, 1‐acetylpiperidine, and tryptophanamide.

## 4 CONCLUSIONS

In this study, a novel technique was explored for the evaluation of seafood products for decomposition. By using sensory data as a training factor, the mass spectral data can be modeled to generate a finding which is more comparable to sensory as compared to alternative chemical analysis techniques. This may provide a valuable compliment to sensory testing in regulatory or industrial settings in the future.

As a qualitative technique, the finding of only a single false negative and positive each out of the 550 test samples demonstrates results quite comparable to those obtained through sensory analysis. Reproducibility via triplicate measurements across the decomposition range on separate days has also been demonstrated.

By evaluating the predictive power of compounds of interest, it was possible to identify compounds which are indicative of decomposition in these products and have not previously been explored for this purpose. These may provide an interesting avenue for future work.

## CONFLICT OF INTEREST

The authors declare no conflict of interest.

## Data Availability

Due to the very large quantity of data, this will be made available upon request.

## References

[fsn32223-bib-0001] Anastassiades, M. , Lehotay, S. J. , Stajnbaher, D. , & Schenck, F. J. (2003). Fast and easy multiresidue method employing acetonitrile extraction/partitioning and dispersive solid‐phase extraction for the determination of pesticide residues in produce. Journal of AOAC International, 86(2), 412–431. 10.1093/jaoac/86.2.412 12723926

[fsn32223-bib-0002] AOAC . (1982). Indole in shrimp, liquid chromatographic fluorometric method. AOAC Official Methods of Analysis, 20(981.07).

[fsn32223-bib-0003] AOAC . (1987). Histamine in seafood fluorometric method. AOAC Official Methods of Analysis, 20(977.13).

[fsn32223-bib-0004] ASTM . (1981). Guidelines for the Selection and Training of Sensory Panel Members. In (pp. 1). ASTM International.

[fsn32223-bib-0005] Bai, J. , Baker, S. M. , Goodrich‐Schneider, R. M. , Montazeri, N. , & Sarnoski, P. J. (2019). Aroma profile characterization of Mahi‐Mahi and Tuna for determining spoilage using purge and trap gas chromatography‐mass spectrometry. Journal of Food Science, 84(3), 481–489. 10.1111/1750-3841.14478 30775780

[fsn32223-bib-0006] Breiman, L. (2001). Random forests. Machine Learning, 45(1), 5–32. 10.1023/a:1010933404324

[fsn32223-bib-0007] Bunch, E. A. , Altwein, D. M. , Johnson, L. E. , Farley, J. R. , & Hammersmith, A. A. (1995). Homogeneous sample preparation of raw shrimp using dry ice. Journal of AOAC International, 78(3), 883–887. 10.1093/jaoac/78.3.883 7756906

[fsn32223-bib-0008] Chang, O. , Cheuk, W. L. , Nickelson, R. , Martin, R. , & Finne, G. (1983). Indole in shrimp: Effect of fresh storage temperature, freezing and boiling. Journal of Food Science, 48(3), 813–816. 10.1111/j.1365-2621.1983.tb14906.x

[fsn32223-bib-0009] Du, W.‐X. , Huang, T.‐S. , Kim, J. , Marshall, M. R. , & Wei, C.‐I. (2001). Chemical, microbiological, and aromascan evaluation of Mahi‐Mahi fillets under various storage conditions. Journal of Agricultural and Food Chemistry, 49(1), 527–534. 10.1021/jf0011135 11305257

[fsn32223-bib-0010] Emborg, J. , Laursen, B. G. , Rathjen, T. , & Dalgaard, P. (2002). Microbial spoilage and formation of biogenic amines in fresh and thawed modified atmosphere‐packed salmon (Salmo salar) at 2°C. Journal of Applied Microbiology, 92(4), 790–799. 10.1046/j.1365-2672.2002.01588.x 11966922

[fsn32223-bib-0011] Fan, X. , Lin, X. , Wu, C. , Zhang, N. , Cheng, Q. , Qi, H. , Konno, K. , & Dong, X. (2021). Estimating freshness of ice storage rainbow trout using bioelectrical impedance analysis. Food Science & Nutrition, 9(1), 154–163. 10.1002/fsn3.1974 33473279 PMC7802552

[fsn32223-bib-0012] FAO, F. a. A. O. o. t. U. N.‐ . (1999). Guidelines for the sensory evaluation of fish and shellfish in laboratories. Codex Alimentarius, 31, 1–32.

[fsn32223-bib-0013] Faullimel, C. , Ennahar, S. , Aoude‐Werner, D. , Guterl, P. , & Marchioni, E. (2005). DNA comet assay for the detection of time‐temperature abuse during the storage of poultry. Journal of Food Protection, 68(7), 1414–1420. 10.4315/0362-028x-68.7.1414 16013379

[fsn32223-bib-0014] Guijas, C. , Montenegro‐Burke, J. R. , Domingo‐Almenara, X. , Palermo, A. , Warth, B. , Hermann, G. , … Siuzdak, G. (2018). METLIN: A technology platform for identifying knowns and unknowns. Analytical Chemistry, 90(5), 3156–3164. 10.1021/acs.analchem.7b04424 29381867 PMC5933435

[fsn32223-bib-0015] HighChem LLC . (2020). mzCloud. Retrieved from mzcloud.org

[fsn32223-bib-0016] Hungerford, J. M. (2010). Scombroid poisoning: A review. Toxicon, 56(2), 231–243. 10.1016/j.toxicon.2010.02.006 20152850

[fsn32223-bib-0017] Jaffrès, E. , Lalanne, V. , Macé, S. , Cornet, J. , Cardinal, M. , Sérot, T. , Dousset, X. , & Joffraud, J.‐J. (2011). Sensory characteristics of spoilage and volatile compounds associated with bacteria isolated from cooked and peeled tropical shrimps using SPME–GC–MS analysis. International Journal of Food Microbiology, 147(3), 195–202. 10.1016/j.ijfoodmicro.2011.04.008 21531471

[fsn32223-bib-0018] Joffraud, J. J. , Leroi, F. , Roy, C. , & Berdagué, J. L. (2001). Characterisation of volatile compounds produced by bacteria isolated from the spoilage flora of cold‐smoked salmon. International Journal of Food Microbiology, 66(3), 175–184. 10.1016/S0168-1605(00)00532-8 11428576

[fsn32223-bib-0019] Jørgensen, L. V. , Huss, H. H. , & Dalgaard, P. (2001). Significance of volatile compounds produced by spoilage bacteria in vacuum‐packed cold‐smoked salmon (Salmo salar) analyzed by GC‐MS and multivariate regression. Journal of Agricultural and Food Chemistry, 49(5), 2376–2381. 10.1021/jf0009908 11368607

[fsn32223-bib-0020] Kanehisa, M. , & Goto, S. (2000). KEGG: Kyoto encyclopedia of genes and genomes. Nucleic Acids Research, 28(1), 27–30. 10.1093/nar/28.1.27 10592173 PMC102409

[fsn32223-bib-0021] Kuuliala, L. , Abatih, E. , Ioannidis, A. G. , Vanderroost, M. , De Meulenaer, B. , Ragaert, P. , & Devlieghere, F. (2018). Multivariate statistical analysis for the identification of potential seafood spoilage indicators. Food Control, 84, 49–60. 10.1016/j.foodcont.2017.07.018

[fsn32223-bib-0022] Labbe, D. , Rytz, A. , & Hugi, A. (2004). Training is a critical step to obtain reliable product profiles in a real food industry context. Food Quality and Preference, 15(4), 341–348. 10.1016/S0950-3293(03)00081-8

[fsn32223-bib-0023] Leroi, F. , Joffraud, J. J. , Chevalier, F. , & Cardinal, M. (2001). Research of quality indices for cold‐smoked salmon using a stepwise multiple regression of microbiological counts and physico‐chemical parameters. Journal of Applied Microbiology, 90(4), 578–587. 10.1046/j.1365-2672.2001.01283.x 11309070

[fsn32223-bib-0024] Lim, J. H. , Park, J. , Ahn, J. H. , Jin, H. J. , Hong, S. , & Park, T. H. (2013). A peptide receptor‐based bioelectronic nose for the real‐time determination of seafood quality. Biosensors and Bioelectronics, 39(1), 244–249. 10.1016/j.bios.2012.07.054 22901715

[fsn32223-bib-0025] Lv, R. , Huang, X. , Aheto, J. H. , Mu, L. , & Tian, X. (2018). Analysis of fish spoilage by gas chromatography–mass spectrometry and electronic olfaction bionic system. Journal of Food Safety, 38(6), e12557. 10.1111/jfs.12557

[fsn32223-bib-0026] Mikš‐Krajnik, M. , Yoon, Y.‐J. , Ukuku, D. O. , & Yuk, H.‐G. (2016). Volatile chemical spoilage indexes of raw Atlantic salmon (Salmo salar) stored under aerobic condition in relation to microbiological and sensory shelf lives. Food Microbiology, 53, 182–191. 10.1016/j.fm.2015.10.001 26678146

[fsn32223-bib-0027] Næs, T. (1990). Handling individual differences between assessors in sensory profiling. Food Quality and Preference, 2(3), 187–199. 10.1016/0950-3293(90)90023-N

[fsn32223-bib-0028] Rossi, F. (2001). Assessing sensory panelist performance using repeatability and reproducibility measures. Food Quality and Preference, 12(5), 467–479. 10.1016/S0950-3293(01)00038-6

[fsn32223-bib-0029] Royal Society of Chemistry . (2020). ChemSpider. Retrieved from www.chemspider.com

[fsn32223-bib-0030] SANCO . (2013). Guidance document on analytical quality control and validation procedures for pesticide residues analysis in food and feed. SANCO/12571.

[fsn32223-bib-0031] Self, R. L. , McLendon, M. G. , & Lock, C. M. (2019). Determination of decomposition in Salmon products by mass spectrometry with sensory‐driven multivariate analysis. Journal of Food Safety, 39(5), e12676. 10.1111/jfs.12676

[fsn32223-bib-0032] Self, R. L. , Wu, W.‐H. , & Marks, H. S. (2011). Simultaneous quantification of eight biogenic amine compounds in tuna by matrix solid‐phase dispersion followed by HPLC–orbitrap mass spectrometry. Journal of Agricultural and Food Chemistry, 59(11), 5906–5913. 10.1021/jf200455r 21534596

[fsn32223-bib-0033] Sun, Z. , Liang, L. , Li, J. , Liu, X. , Sun, J. , Zou, X. , Zuo, M. , & Guo, Z. (2020). Rapid detection of Atlantic salmon multi‐quality based on impedance properties. Food Science & Nutrition, 8(2), 862–869. 10.1002/fsn3.1362 32148795 PMC7020269

[fsn32223-bib-0034] U. S. Congress . (2018). Explanatory statement submitted by mr. frelinghuysen, chairman of the house committee on appropriations, regarding the house amendment to senate amendment on H.R. 1625. Congressional Record, 164(50), H2051.

[fsn32223-bib-0035] USFDA . (2010). Compliance program guidance manual (7303.844). US Food and Drug Admin.

[fsn32223-bib-0036] USFDA . (2013). Sensory Analysis. In ORA Laboratory Manual (Vol. 4). White Oak, MD.

[fsn32223-bib-0037] Wilkinson, C. , & Yuksel, D. (1997). Modeling differences between panelists in use of measurement scales. Journal of Sensory Studies, 12(1), 55–68. 10.1111/j.1745-459X.1997.tb00053.x

